# Step ladder VY advancement flap for lower eyelid reconstruction after resection eyelid malignant tumors

**DOI:** 10.1007/s10792-024-03203-9

**Published:** 2024-06-29

**Authors:** Kento Yamashita, Kazuki Shimada, Kohei Aoki, Norihito Ito, Takako Komiya, Yukiko Ida, Yoshihiko Usui, Hiroshi Goto, Hajime Matsumura

**Affiliations:** 1 Department of Plastic and Reconstructive Surgery, Tokyo Medical University, 6-7-1 Nhishisinjyuku Shinjyuku-Ku, Tokyo, 160-0023 Japan; 2 Department of Ophthalmology, Tokyo Medical University, Tokyo, Japan

**Keywords:** Step ladder, VY advancement flap, Lower eyelid, Eyelid reconstruction

## Abstract

**Background:**

In oculoplastic surgery, reconstruction of a large defect after the removal of a massive malignant lower lid tumor still represents a unique challenge. We will report on this case, including a presentation of the case using step ladder V–Y advancement flap.

**Methods:**

During November 2018 to March 2023, five patients of lower eyelid malignant tumor had wide resection with safety margin and reconstructed using step ladder V–Y advancement flap. The flap was used step ladder V–Y advancement flap.

**Results:**

No complications, including ectropion deformity, occurred. This flap does not sacrifice healthy skin as seen with the cheek rotation flap, and the area of dissection is very small and can be performed in a short time.

**Conclusions:**

Step ladder V–Y advancement flap is highly useful in cases that require a reconstruction of a large defect after the removal of a massive malignant lower lid tumor from viewpoints of operating time, ease of procedure, aesthetics, and complications.

## Introduction

The eyelid is a highly anatomically complex region that serves as a shield that protects the eye and forms and maintains a lacrimal film.

Malignant tumors that arise in the eyelid and conjunctiva include basal cell carcinoma squamous cell carcinoma (SCC), sebaceous adenocarcinoma, and melanoma.

These tumors must be controlled, and the goal of oncologic surgery in this area is complete resection of the tumor lesion, with reconstruction also being important for functional and cosmetic reasons [1]. To repair large defects in both the lower and upper eyelids, it's generally assumed that one layer should be fixed with a blood-supplied flap to ensure the eyelid's survival, while the other layer can be patched with a separate graft. The most common method in ophthalmology area to fix the lower eyelid is the modified Hughes technique [2]. This method uses a flap from the upper eyelid for the inner layer and a skin graft for the outer layer. For upper eyelid defects, the Cutler-Beard method is often applied, where a full-thickness flap from the lower eyelid is moved up behind a preserved lower eyelid edge. Moreover, several techniques can be used to reconstruct lower eyelid defects [3–6].

Posterior lamellae reconstruction with oral and nasal mucosa [7, 8] and anterior lamellae reconstruction with a cheek rotation flap (Mustardé flap) are the most common procedures for full and wide defects of the lower eyelid [9].

However, this approach involves excision of the healthy skin of the buccal area, which is a significant sacrifice.

In this study, we present here a clinical series of 5 malignant eyelid tumor patients reconstructed a wide anterior lamellae defect of the lower eyelid by moving the step ladder V–Y advancement flap of the buccal area obliquely upward after full and wide defects of the lower eyelid. This is an excellent method in terms of function and cosmetic appearance, with little sacrifice of healthy skin, and the procedu re is relatively easy and can be performed in a short time. Case series is presented.

## Materials and methods

### Patients

During November 2018 to March 2023, five patients of lower eyelid malignant tumor had wide resection with safety margin and reconstructed using step ladder V–Y advancement flap.

Details of the cases including primary disease, defect size, complications, follow-up period are shown in Table 1.

**Table 1:** Characteristics of patients presented in the case series

Case	Age gender	Tumor	Skin defect (mm)	Complications	Recurrence	Follow up
1	83 female	Sebaceous carcinoma	30 × 20	(–)	(–)	5Y1 M
2	51 female	Melanoma	30 × 18	(–)	(–)	2Y9 M
3	78 female	Sebaceous carcinoma	12 × 7	(–)	(–)	2Y3 M
4	57 female	SCC	28 × 17	(–)	(–)	1Y3 M
5	29 male	Melanoma	30 × 7	(–)	(–)	9 M

The postoperative follow-up period ranged from 9 months to 5 year and 1 month, with an average of 29 months.

The individual in this manuscript provided a written informed consent for images obtained and for publication of these case details. As this was small case report on the surgical technique, and institutional review board (IRB) approval was not required.

### Surgical technique

After wide resection, surgical margins were ensured intraoperative pathological diagnosis, posterior lamellae was reconstructed using palatal mucosa graft. If there is a defect in the bulbar conjunctiva beyond the conjunctival fornix, it is taken from the ipsilateral upper bulbar conjunctiva; if not enough, it is taken from the contralateral bulbar conjunctiva; and is free grafted. If the conjunctival fornix is not exceeded and only the bulbar conjunctiva is defective, the palatal mucous is free grafted.

The pretarsal part was excised together with the tumor. After resection of the tumor, if the tumor is relatively small and preseptal part remained after excision, preseptal part and the orbital part of the orbicularis oculi muscle were included in the skin flap. If the preseptal part was resected with the tumor, the orbital part of the orbicularis oculi muscle was included in the skin flap.

For anterior lamellae reconstruction, step ladder V–Y advancement flap from buccal area was used. The flap was elevated along with the underlying orbicularis oculi muscle.

The width of the flap should be the same length or little wider as the defect, and the vertical length should be larger than the defect for one step, slightly smaller for the second step, and even smaller for the third step. When the vertical length of the skin defect is large and requires a longer distance for the flap to travel, a fourth step may be added. The flap moves slightly inward and upward.

There are two key points in this technique. As the first point, the flap is not positioned so that it does not exceed the esthetic unit. In particular, avoid entering the nose area.

The second point is the suture line to be diagonal. The suture line should be designed so that it does not run perpendicular to the relaxed skin tension line (RSTL). When suturing the lower edge of the flap, it should be aligned with the RSTL.

The surrounding area of the flap is elevated from the underlying fascia and the orbicularis oculus in the center of the flap is used as a pedicle and blood flow is maintained. Blood flow in the skin flap is very good by attaching the orbicularis oculus, and adequate mobilization can be achieved.

## Results

In all cases, the defects were successfully covered without any blood flow impairment, and primary wound closure was obtained. Recurrence was not evident within the follow-up period.

Notably, there was no complication, including ectropion and the cosmetic outcome was excellent in all cases.

### Case presentation

**Fig. 1:**
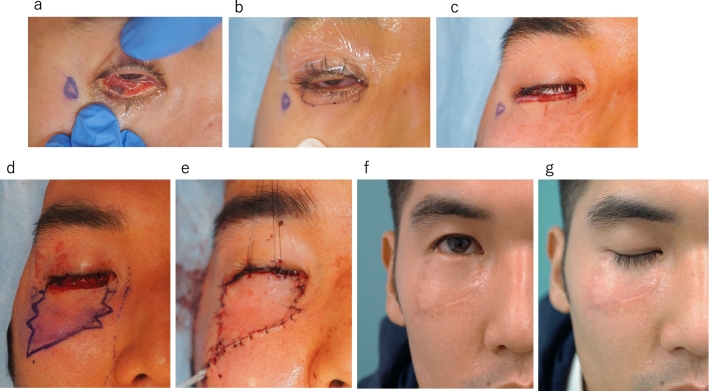
Case5 **a** Nodular tumor with sporadic melanosis involving the lateral half of the right lower lid, **b** Resection area, **c** After resection, **d** Flap design, **e** Elevated flap image, **f** After suturing, **g** 1 year after surgery, opening eyelid, **h** closed eyelid

Case 5 29-year-old male (Fig. 1).

He was aware of a tumor on his right lower eyelid and consulted the ophthalmology department of his local doctor. He was referred to ophthalmology clinic at Tokyo Medical University Hospital. for a consultation. At the time of his initial visit, the tumor was non-pigmented and showed a tendency to shrink with steroid eye drops. However, it gradually became pigmented and tended to increase in size, so a biopsy was performed. A diagnosis of malignant melanoma was confirmed. The patient was referred to our department because melanosis was also present in the conjunctiva of the eyelid and the size of the tumor indicated that reconstruction of the lower eyelid was necessary.

The skin defect after tumor resection was 30 mm × 7 mm, and the conjunctival defect was 30 mm × 10 mm. A 30 mm × 10 mm piece of mucosa was harvested from the hard palate and grafted onto the conjunctiva. The skin and eyelid margin were reconstructed with a Step Ladder V–Y Advancement Flap (2 Step) from the buccal area. The eyelids were able to close six months after surgery, and the results were very satisfactory both functionally and cosmetically.

## Discussion

The skin defect after eyelid tumor resection is not an inverted triangle, but a horizontal rectangle in most cases. This skin defect shape may be due to the reason that many lower eyelid malignancies originate at the eyelid margin [10].

In turn, resection with a safety margin often results in a rectangular or trapezoidal defect that includes the eyelid margin.

For this type of anterior lamellae tissue defect involving the lower eyelid margin, reconstruction by cheek rotation flap is commonly performed [9]. However, when a cheek rotation flap is used, the healthy skin tissue below the tissue defect is usually excised with wide resection to correct the dog ear deformity. Furthermore, a large skin flap must be elevated and rotated from the entire buccal area, including the outer ocular area and anterior ear area. Therefore, it must be said that reconstruction with a cheek rotation flap is relatively invasive.

Moving the cheek skin upward below the defect with a V–Y advancement lower eyelid flap is relatively easier and quicker to perform than a cheek rotational skin flap [11]. And then, because subcutaneous pedicle of V–Y flap contains direct cutaneous perforators as opposed to the random pattern of the cheek rotation flap, it has a better blood supply [11].

However, in this case, the two longitudinal oblique scars are likely to cause the skin flap to droop due to contraction and gravity, resulting in an ectropion formation [11]. In addition, the triangular scar is more likely to cause trapdoor deformity, and the scar is also more visible because the incision line intersects the RSTL [12].

To solve this problem, we developed a reconstruction method using a Step ladder V–Y advancement flap. The step ladder V–Y advancement flap is a skin flap commonly used for hand and foot reconstruction [13–15]. However, as far as we could find, there are no reports of its use in lower eyelid reconstruction.

The step ladder shape minimizes drooping of the skin flap, and by making the first step slightly lengthwise-wider than the defect, a sufficient amount of tissue can be placed at the eyelid margin to prevent ectropion. In fact, no cases of ectropion occurred. Furthermore, moving the direction of the flap slightly more oblique than vertical can also minimize drooping, because the flap is less likely to be pulled downward.

On the other hand, this flap is inferior to the cheek rotation flap in some respect. In the cheek rotation flap, the scar is only noticeable in the outer ocular area [9]; in the step ladder V–Y advancement flap, the V-shaped scar is in the front of the face. However, because of the zig-zag incision of the step ladder, the scar is not a problem, as shown in the case presentation.

The most significant disadvantage of this flap over the cheek rotation flap is that lymphatic dissection of the parotid region cannot be performed in the same surgical field at the same time.

In conclusion, reconstruction with a Step ladder V–Y advancement flap is very useful for anterior lamellae defects of the lower eyelid. This technique provides a very satisfactory cosmetic result without sacrificing healthy tissue. In addition, the operative time is reduced.

## References

[1] Yano T, Karakawa R, Tomoyoshi Shibata I et al (2021) Ideal esthetic and functional full-thickness lower eyelid “like with like” reconstruction using a combined Hughes flap and swing skin flap technique. J Plast Reconstr Aesthet Surg 74:3015–3021 34023240 10.1016/j.bjps.2021.03.119

[2] Hughes WL (1941) Reconstruction of the eyelids. Trans Am Ophthalmol Soc 39:437 16693264 PMC1315024

[3] Sayag D, Ducasse A, Coicaud C et al (2001) Tarsomarginal graft. Indication and results in palpebral surgery. J Fr Ophthalmol 24(7):724–728 11591912

[4] Hawes MJ (2013) Free autogenous grafts in eyelid tarsoconjunctival reconstruction. Ophthalmic Surg Lasers Imaging Retina 18(1):37–41 3561935

[5] Paridaens D, van den Bosch WA (2008) Orbicularis muscle advancement flap combined with free posterior and anterior lamellar grafts: a one-stage “sandwich” technique for eyelid reconstruction. Ophthalmology 115(1):189–194 17559937 10.1016/j.ophtha.2007.03.022

[6] Tenzel RR, Stewart WB (1978) Eyelid reconstruction by semicircular flap technique. Ophthalmology 85(11):1164–1169 733166 10.1016/s0161-6420(78)35578-0

[7] Siegel RJ (1985) Palatal grafts for eyelid reconstruction. Plast Reconstr Surg 76:411–414 4034758 10.1097/00006534-198509000-00013

[8] Miyamoto J, Nakajima T, Nagasao TI et al (2009) Full-thickness recon- struction of the eyelid with rotation flap based on orbicularis oculi muscle and palatal mucosal graft: long-term results in 12 cases. J Plast Reconstr Aesthet Surg 62:1389–1394 18784003 10.1016/j.bjps.2008.05.040

[9] Callahan MA, Callahan A (1980) Mustardé flap lower lid reconstruction after malignancy. Ophthalmology 87(4):279–286 7393532 10.1016/s0161-6420(80)35237-8

[10] Pickford MA, Hogg FJ, Fallowfield ME et al (1995) Sebaceous carcinoma of the periorbital and extraorbital regions. Br J Plastic Surg 28:93–96 10.1016/0007-1226(95)90103-5 7743054

[11] Sugg KB, Cederna PS, Brown DL (2013) The V-Y advancement flap is equivalent to the Mustardé flap for ectropion prevention in the reconstruction of moderate-size lid-cheek junction defects. Plast Reconstr Surg 131(1):28e–36e 23271551 10.1097/PRS.0b013e3182729e22 PMC4487805

[12] Waleed G, Anne HJ, James G et al (2019) Lower eyelid reconstruction using a nasolabial, perforator-based V-Y advancement flap expanding the utility of facial perforator flaps. Ann Plast Surg 82(1):46–52 30113981 10.1097/SAP.0000000000001576

[13] Hayashi A, Maruyama Y (1997) Stepladder V-Y advancement flap for repair of postero-plantar heel ulcers. Br J Plast Surg 50:657–661 9613413 10.1016/s0007-1226(97)90516-6

[14] Furubayashi G, Sawaizumi M, Maeda MI et al (2017) Plantar reconstruction using a step-ladder advancement flap. JPRAS Open 13:41–45

[15] Evans DM, Martin DL (1988) Step-advancement island flap for fingertip reconstruction. Br J Plast Surg 41:105–111 3349214 10.1016/0007-1226(88)90035-5

